# Comparative functional analysis of papaya leaves at different developmental stages

**DOI:** 10.5511/plantbiotechnology.25.0616a

**Published:** 2025-12-25

**Authors:** Kota Kera, Kosuke Soma, Nanami Sugimoto, Haruna Inoue, Akihito Endo, Masumi Iijima, Hideyuki Suzuki

**Affiliations:** 1 Department of Nutritional Science and Food Safety, Faculty of Applied Bioscience, Tokyo University of Agriculture, 1-1-1 Sakuragaoka, Setagaya-ku, Tokyo 156-8502, Japan; 2 Department of Research and Development, Kazusa DNA Research Institute, 2-6-7 Kazusa-Kamatari, Kisarazu, Chiba 292-0818, Japan

**Keywords:** *Carica papaya* L., carpaine, dehydrocarpaine, γ-aminobutyric acid, metabolome analysis

## Abstract

Papaya (*Carica papaya* L.), a tropical plant belonging to the *Caricaceae* family, is widely cultivated in tropical and subtropical countries. Young leaves grow from the stem tips, petioles elongate, and leaf color changes from light green to dark green during development. Papaya leaves are used as therapeutic agents in folk medicine and potential functional food materials; however, the specific associations between the leaf development stage and functional components of papaya remain unknown. Therefore, in this study, we aimed to conduct a non-targeted analysis of the four developmental stages of papaya leaves via liquid chromatography coupled with quadrupole-time-of-flight mass spectrometry. Specifically, we focused on carpaine derivatives and γ-aminobutyric acid that have attracted attention in the Japanese functional food industry. Carpaine derivatives were abundant in young leaves; however, their levels decreased with increasing leaf maturity. In contrast, γ-aminobutyric acid levels increased with increasing leaf maturity. Multivariate analyses revealed that the metabolites changed more significantly during the transition to the dark green phase than during the transition from the early yellow green to bright green phase. Additionally, proteolytic activity was evaluated using casein as a substrate. Proteolytic activity decreased with increasing leaf maturity. In conclusion, our findings suggest that leaves at different developmental stages should be selected based on their functional components and intended application.

Papaya (*Carica papaya* L.) is a tropical plant belonging to the *Caricaceae* family that is widely cultivated in tropical and subtropical countries. It is a perennial herbaceous plant that can grow to over 10 m in height for nearly a decade (Silva et al. 2007). Young leaves arise from the stem tips, petioles elongate over time, and leaf color changes from light to dark green (Figure 1). Asian countries, such as Malaysia, the Philippines, and India, traditionally consume papaya leaves as vegetables or drink them as tea (Ikram et al. 2015). In addition to their use in food, papaya leaves are applied as therapeutic agents in folk medicine. Papaya leaves also protect against diabetes, high blood pressure, digestive disorders, cancer, inflammation, dengue fever, malaria, parasitic infectins, and severe acute respiratory syndrome-coronavirus-2 infection (Adel et al. 2022; Ikram et al. 2015; Jaiswal and Jain 2018). These physiological activities are possibly due to their constituent phytochemicals, including alkaloids such as carpaine (Julianti et al. 2014a, b; Zunjar et al. 2016), saponins (Vuong et al. 2013), and phenolic compounds (Canini et al. 2007; Chaijan et al. 2024; Gogna et al. 2015; Misnan et al. 2024; Vuong et al. 2013). In addition to these functional ingredients, papaya leaves contained γ-aminobutyric acid (GABA), which has recently attracted attention in the Japanese functional food industry (Gogna et al. 2015). Interestingly, carpaine and phenolic compound levels vary across different leaf developmental stages (Gogna et al. 2015; Julianti et al. 2014b; Misnan et al. 2024; Patil 2024). In this study, we performed non-targeted analysis of the four developmental stages of papaya leaves using liquid chromatography coupled with quadrupole-time-of-flight mass spectrometry (LC/Q-TOF MS) and examined the variations in the levels of carpaine, its derivatives, and GABA at different development stages to contribute to the effective use of papaya leaves in functional food.

**Figure 1:**
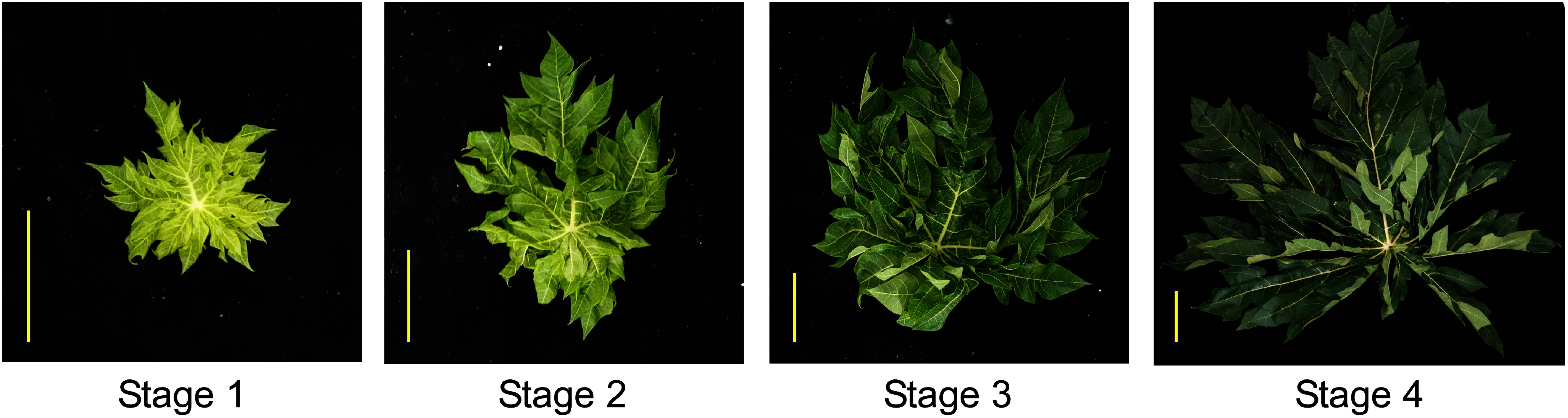
Figure 1. Photographs of the papaya leaves. Typical papaya leaves at different developmental stages. Stage 1, young newly expanded leaves with a light green color; stage 2, young slightly enlarged tender leaves with a light green color; stage 3, mature leaves with a bright green color; stage 4, fully mature slightly hardened leaves with a dark green color. Scale bars, 10 cm.

Papaya trees, “Sun-papaya”, were grown in open fields at Yaginuma Farm Co., Ltd. (Ibaraki, Japan). Papaya leaves were obtained in mid-October, 2023 at a temperature of 21°C. First we selected four different female trees and then leaves at different stages of development were collected from each tree according to the following criteria: stage 1, young newly expanded leaves with a light green color; stage 2, young slightly enlarged tender leaves with a light green color; stage 3, mature leaves with a bright green color; stage 4, fully mature slightly hardened leaves with a dark green color. In addition, young leaves were collected near the stem tip and mature leaves were collected from the middle of stem or lower part. The samples without petiole were stored at −80°C. After lyophilization, the leaves were crushed in a milser (IFM-800DGM; Iwatani Corporation, Osaka, Japan), and 10 mg of the resulting powder was mixed with 500 µl of 80% methanol. Subsequently, the supernatant obtained via centrifugation at 15,000 rpm for 10 min was passed through the Mono-Spin C18 column (GL Science Inc., Tokyo, Japan). The eluted solutions were filtered through a 0.2 µm polytetrafluoroethylene membrane (Merck Millipore, MA, USA) and analyzed via LC/Q-TOF MS (Agilent 6530 Accurate Mass Q-TOF; Agilent Technologies, Inc., CA, USA). Analytical conditions were as previously described (Kera et al. 2024). Sample concentrations were adjusted based on the powder dry weight. The sample solution (1 µl) was injected into the InertSustain AQ-C18 column (column size: 2.1×150 mm; particle size: 3.0 µm; GL Science Inc.). Mobile phases A (0.1% (v/v) formic acid in water) and B (0.1% (v/v) formic acid in acetonitrile) were used, with a gradient of 2% (v/v) B for 0–3 min, 2–98% (v/v) B for 3–30 min, 98% (v/v) B for 30–35 min, and 2% (v/v) B for 35–40 min. The column temperature and flow rate were maintained at 40°C and 200 µl min^−1^, respectively. Mass spectrometry analysis was performed in the electrospray ionization positive mode, covering a mass range of *m*/*z* 50–1,500 for full mass scans. An automated processing program based on ProteoWizard (Chambers et al. 2012) and PowerGet (Sakurai et al. 2014) was used for peak detection, characterization, and alignment. Metabolite annotation was performed using MFSearcher (Sakurai et al. 2013), with a mass accuracy of 5–20 ppm. Carpaine and GABA were identified by comparing with the authentic compounds purchased from Biorbyt (Cambridge, UK) and Tokyo Chemical Industry Co. Ltd. (Tokyo, Japan), respectively. Other compounds were annotated based on predictions from database searches and previous reports (Hiraga et al. 2021; Kera et al. 2024). The resulting peak list (Supplementary Dataset1) was generated, and further multivariate analyses were performed using SIMCA-P (version 17.0.2, Sartorius Stedim Biotech S.A., Aubagne, France).

First, we focused on carpaine and its derivatives, dehydrocarpaine I and dehydrocarpaine II, which are *Caricaceae* family-specific major piperidine alkaloids (Julianti et al. 2014b), and analyzed the variations in their levels during leaf development. Their peak areas (carpaine: *m*/*z* 240.1958±20 ppm [M+2H]^2+^; dehydrocarpaine I: *m*/*z* 477.3687±20 ppm [M+H]^+^; dehydrocarpaine II: *m*/*z* 475.3530±20 ppm [M+H]^+^) were determined using the MassHunter software version B.05.01 (Agilent Technologies, Inc.; Figure 2). Calibration curves of carpaine were plotted in the concentration range of 0.5–10 µM. Carpaine levels showed little difference in stages 1–3 but declined in stage 4. Dehydrocarpaine I and Dehydrocarpaine II levels were determined as carpain equivalents. Dehydrocarpaine I levels were lower in stage 4 than in stage 2; however, its levels in stages 1 and 3 were not significantly different from those in stage 4. Similarly, dehydrocarpaine II levels were lower in stage 4 than in stage 1; however, its levels in stages 2 and 3 were not significantly different from those in stage 4. Therefore, carpaine and dehydrocarpaines I and II were abundant in young leaves, but their levels decreased with increasing leaf maturity. The analyzed articles were divided based on the relationship between the leaf maturity stage and carpaine levels. Studies from the first half of the 20th century indicated that young leaves contain 3–4-times more carpaine than the older leaves (Barger et al. 1937). In contrast, Julianti et al. (2014b) compared the young and old leaves of the same tree and concluded that carpaine levels were not related to the leaf age. Yap et al. (2021) also reported that young leaves contain approximately 1.2–1.5-times more carpaine than the old leaves. One possible reason for this difference is the ambiguity in the definitions of young and old leaves. Julianti et al. collected young leaves from the top and old leaves from the lower part of a tree, whereas Yap et al. considered green leaves as young leaves and yellow withered leaves as old leaves. Based on our criteria, the young leaves studied by Julianti et al. corresponded to the stage 1 or 2 leaves and old leaves to the stage 3 or 4 leaves, whereas the young leaves studied by Yap et al. corresponded to the stage 4 leaves in this study. Another possibility is differences in growing environment. The leaves used in previous reports were grown in tropical regions such as Indonesia (Julianti et al. 2014b) and Malaysia (Yap et al. 2021), while our samples were grown in Ibaraki prefecture, Japan, temperate zones. Carpamic acid and dehydrocarpamic acid, putative composition units of carpaine, dehydrocarpaine I, and dehydrocarpaine II, were similarly analyzed. Carpamic acid levels significantly increased in stage 4, showing an opposite trend from the change in carpaine levels (Figure 2). Dehydrocarpamic acid levels were lower in stage 4 than in stage 1; however, its levels in stages 2 and 3 were not significantly different from those in stage 4, showing the same changes as dehydrocarpain II levels in stage 2 (Figure 2). The biosynthetic pathway of carpaine and its derivatives is not clear, but carpamic acid may have accumulated in stage 4 as a result of carpaine degradation and accumulation of its precursors.

**Figure 2:**
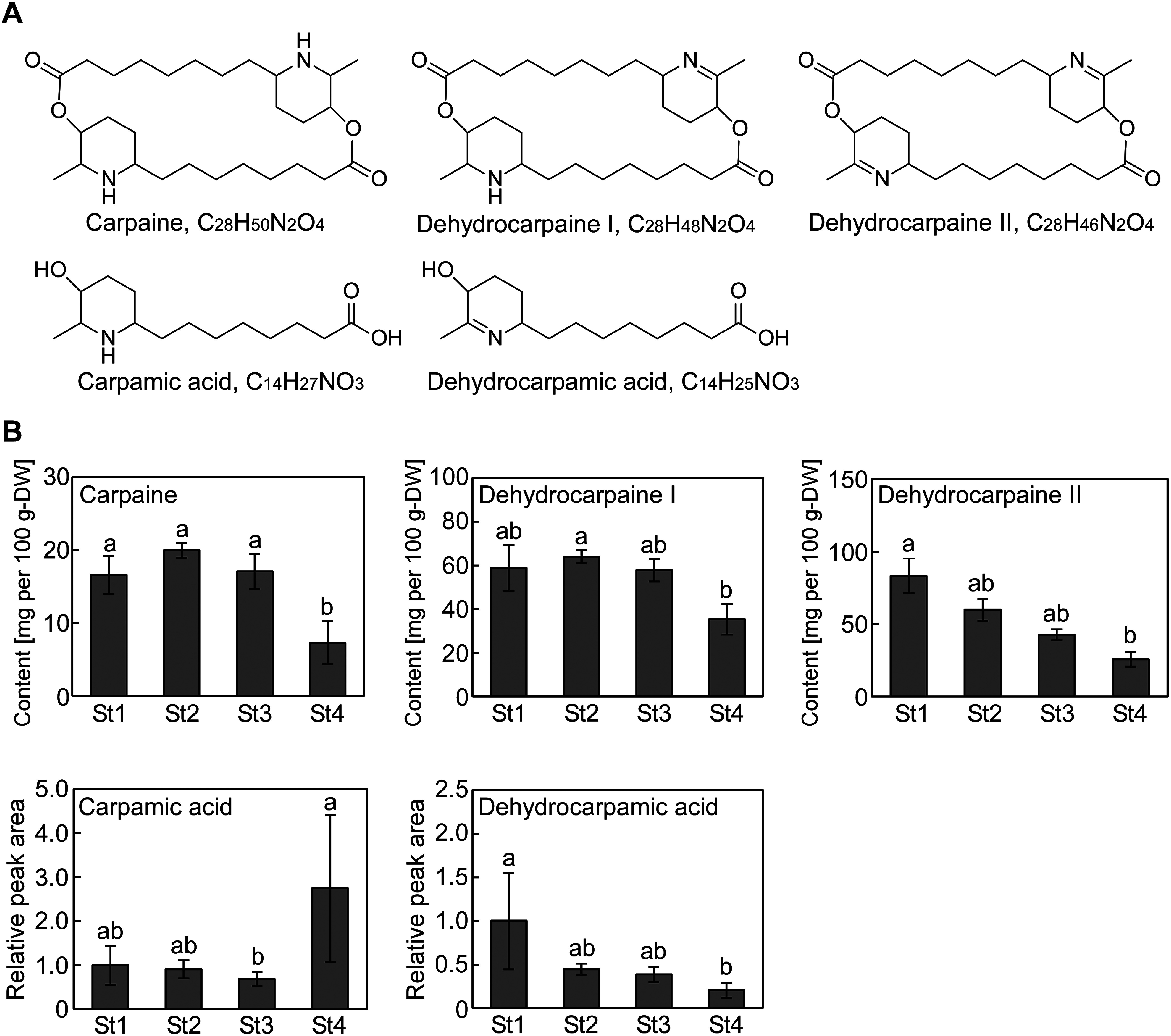
Figure 2. Putative carpaine derivative levels in the papaya leaves. A. Chemical structures of the carpaine derivatives. B. Quantitative carpaine, dehydrocarpaine I, and dehydrocarpaine II levels or relative peak area of carpamic acid and dehydro carpamic acid in the papaya leaves (St1=stage 1, St2=stage 2, St3=stage 3, and St4=stage 4). Dehydrocarpaine I and dehydrocarpaine II levels were determined as carpain equivalents. Values represent the mean±standard deviation (SD) of four biological replicates. Clusters with identical letter codes do not exhibit significant differences, as determined by the Tukey’s test (*p*<0.05).

Levels of GABA, which has attracted attention in the functional food industry, were also quantified in the papaya leaves. Peak area (*m*/*z* 104.0706±20 ppm [M+H]^+^) was determined. Calibration curves were plotted in the concentration range of 25–200 µM. GABA levels were higher in stage 4 than in stages 1 and 3, indicating an increase in GABA levels with increasing leaf maturity (Figure 3). In stage 4, GABA level was 91 mg per 100 g dry weight, and the average GABA level in all stages was 70 mg per 100 g dry weight. GABA levels in common Japanese and Taiwanese green teas are 150 and 56–174 mg per 100 g dry weight, respectively (Tsushida et al. 1987; Wu et al. 2023). Comparatively, GABA levels were not higher in the papaya leaves; however, the levels were not considerably low either, suggesting that these leaves can be used as GABA supplements.

**Figure 3:**
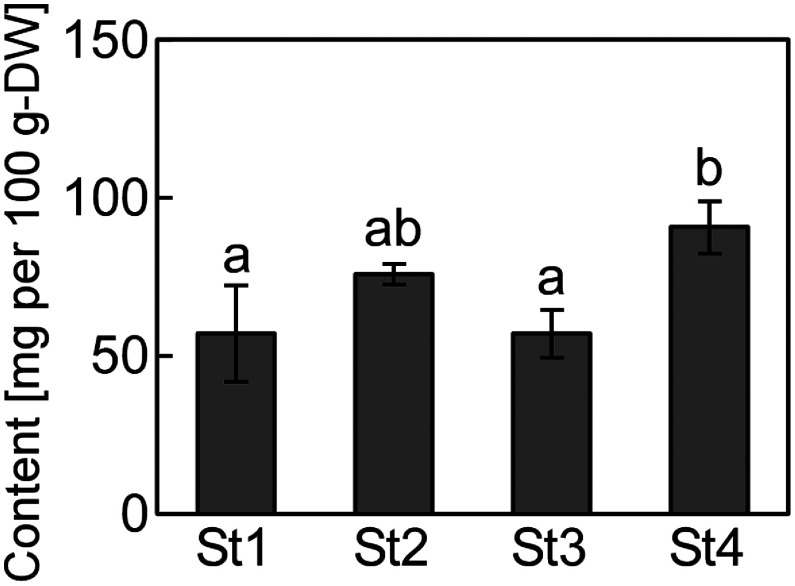
Figure 3. γ-aminobutyric acid (GABA) levels in the papaya leaves. Quantitative GABA levels in the papaya leaves (St1=stage 1, St2=stage 2, St3=stage 3, and St4=stage 4). Values represent the mean±SD of four biological replicates. Clusters with identical letter codes do not exhibit significant differences, as determined by the Tukey’s test (*p*<0.05).

Multivariate analyses were conducted with unit variance using SIMCA-P to assess the metabolite profiles of papaya leaves at different developmental stages. Principal component analysis score plots revealed that one stage 3 sample showed metabolite peak patterns similar to those of stage 4 samples, whereas the other stage 3 samples exhibited metabolite peak patterns similar to those of stage 2 samples (Figure 4A). Moreover, stage 1 samples exhibited metabolite peak patterns similar to those of stage 2 samples, although considerable variation was observed within the groups (Figure 4A). In the loading plot, carpaine derivative levels were significantly high in stages 1, 2, and 3 (Figure 4B). In contrast, GABA was plotted near the center, suggesting not much variation in its levels across different developmental stages (Figure 4B). The peaks with the highest contribution for each stage that were annotated as functional components are also shown in Figure 4B. The alkaloids piperidine and pyrroridine were suggested to contribute significantly to stage 1, possibly related to the accumulation of carpaine derivatives in young leaves. For flavonoids, epigallocatechin was suggested to contribute significantly to stage 1, while kaempferol and quercetin were suggested to contribute significantly to stage 2 and 3. It has been reported that quercetin is more abundant in young leaves than in older leaves (Misnan et al. 2024). Thus, previous report and our results suggest that flavonoids are abundant in yellow-green to bright green leaves. Hierarchical cluster analysis revealed that stages 2 and 3 were the most similar, followed by stages 1 and 4 (Figure 4B). Taken together, principal component and hierarchical cluster analyses indicated that the metabolites changed more significantly during the transition to the dark green phase (stage 4) than during the transition from the yellow green (stage 2) to bright green (stage 3) phase, supporting the aforementioned changes in carpaine derivative levels.

**Figure 4:**
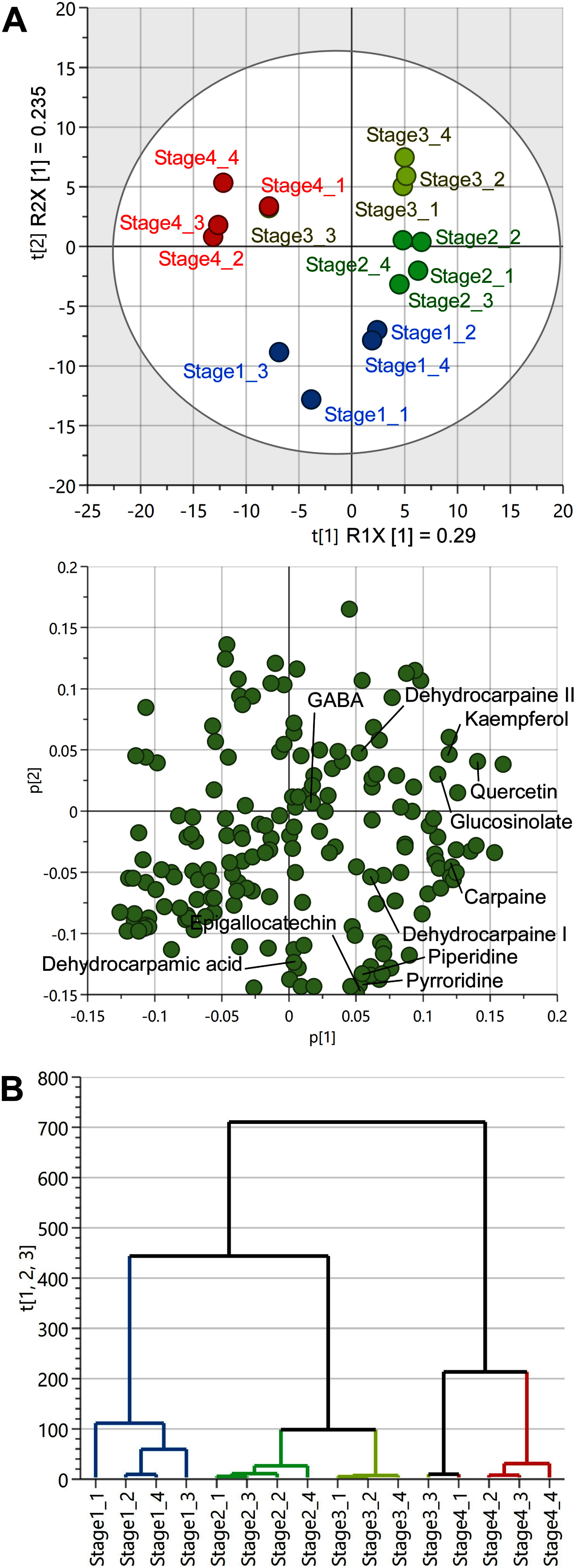
Figure 4. Multivariate analysis of the metabolite profiles of papaya leaves at different developmental stages. A. Principal component analysis score (upper panel) and loading (lower panel) plots. B. Hierarchical cluster analysis dendrogram. The following conditions were used: distance, Euclidean; clustering algorithm, Ward.

Proteolytic activity was determined using casein as a substrate, as previously described (Kera et al. 2024). Lyophilized papaya leaves were suspended in 500 mM phosphate buffer. The supernatant obtained via centrifugation at 18,000×g for 5 min at 4°C was filtered through a 0.45-µm polyvinylidene difluoride membrane (Merck Millipore). The sample solution (20 µl) was mixed with 2.5 µl of 80 mM cysteine solution, 2.5 µl of 40 mM ethylenediaminetetraacetic acid disodium salt solution, and 25 µl of 1% (w/v) casein solution. After reacting at 38°C, the reaction was terminated by adding 75 µl of 5% (w/v) cold trichloroacetic acid solution. Absorbance of the supernatant was measured at 275 nm using a spectrophotometer (U-2900; Hitachi High-Tech Science Corporation, Tokyo, Japan). One unit of proteolytic activity was defined as the enzyme required to increase the absorbance at 275 nm by 0.01 per minute at pH 7.0 and 38°C. Then, protein concentration of the sample solution was determined using the Bradford Protein Assay Kit (Takara Bio Inc., Shiga, Japan). Stage 1 had the highest specific activity of 167 U mg^−1^-protein, which decreased with leaf development to 50 U mg^−1^-protein at stage 4 (Figure 5). The proteolytic activity of unripe papaya fruit peel, pulp, and seeds, which was performed using the same technique, was 280, 54, and 76 U mg^−1^-protein, respectively (Kera et al. 2024). The results suggest that young leaves show better proteolytic activity than the pulp and seeds, although not as good as the peel. Proteolytic enzymes, such as papain, in papaya leaves are used to tenderize meat and purify beverages in the food industry (Esti et al. 2013; Mohd Azmi et al. 2023). Our results suggest that young leaves are more suitable for papaya application in the food industry.

**Figure 5:**
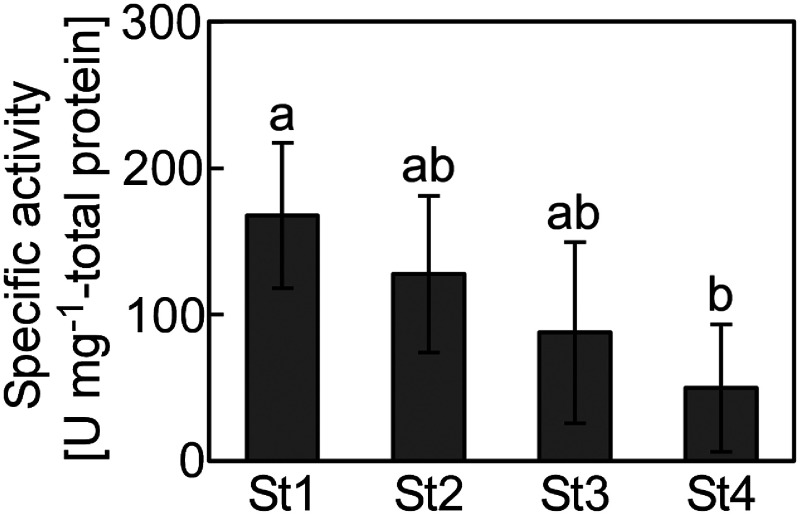
Figure 5. Proteolytic activity of the papaya leaves (St1=stage 1, St2=stage 2, St3=stage 3, and St4=stage 4). Values represent the mean±SD of four biological replicates with three technical replicates. Clusters with identical letter codes do not exhibit significant differences, as determined by the Tukey’s test (*p*<0.05).

In conclusion, although dark green or yellow withered papaya leaves are generally harvested as byproducts of papaya fruit production, this study highlights the superiority of bright green mature leaves in terms of carpaine derivative levels and proteolytic activity and dark green mature leaves in terms of GABA levels.

## Abbreviations

GABA: γ-aminobutyric acid
LC/Q-TOF MS: liquid chromatography coupled with quadrupole-time-of-flight mass spectrometry

## Data deposition

The raw datasets were deposited into the MetaboBank metabolome database under the accession numbers MTBKS260 and are available for free download.

## Description of supplementary files

Supplementary Dataset1. Peak list for papaya leaves.
